# Transport and expression of transporters for 3-O-methyl-D-glucose and L-methionine along the intestine of broiler chickens receiving different methionine supplements^☆^

**DOI:** 10.1016/j.psj.2025.105142

**Published:** 2025-04-07

**Authors:** Julia Riedel, Isabel I. Schermuly, Stella Romanet, Eva-Maria Saliu, Andreas Lemme, Jürgen Zentek, Jörg R. Aschenbach

**Affiliations:** a Institute of Veterinary Physiology, Freie Universität Berlin, Berlin, Germany; b Institute of Animal Nutrition, Freie Universität Berlin, Berlin, Germany; c Evonik Operations GmbH, Animal Nutrition Services, Hanau-Wolfgang, Germany

**Keywords:** Methionine supplementation, 3-O-methyl-d-glucose, Intestinal absorption, Ussing chamber, Broiler

## Abstract

The present study hypothesized that supplementation of different methionine (**Met**) sources might influence the intestinal absorption of l-Met and 3-O-methyl-d-glucose (**3-OMG**) in broilers. In a completely randomized study, a total of 53 Cobb500 broilers (30 males and 23 females) received a grower-finisher diet that was either not supplemented with Met (Met + Cys, 0.49 %; control) or supplemented with either 0.27 % l-Met, 0.27 % DL-Met or 0.47 % DL-2‑hydroxy-4-(methylthio) butanoic acid (**HMTBA**). After ≥10 days on the diets, uptakes of 3-OMG and l-Met were measured in duodenum, mid-jejunum and caecum at 50 µM and 5 mM concentrations in Ussing chambers, each in the presence and absence of Na^+^. We also investigated the mRNA expression of apical glucose and Met transporters. Dietary supplements had no effect on 3-OMG and l-Met uptakes (*P* > 0.05), except for male broilers receiving DL-Met or DL-HMTBA, that showed higher jejunal uptakes of l-Met than control at 5 mM (*P* < 0.001). Except for l-Met uptakes at 5 mM, tissue × sodium interactions (*P* ≤ 0.05) for 3-OMG and l-Met uptakes verified higher uptakes in jejunum compared to duodenum and caecum; with higher uptakes in the presence vs. absence of Na^+^ in jejunum only. In duodenum, uptakes of l-Met and 3-OMG at 50 µM concentration were higher in males vs. females. Expression of SGLT1, B^0^AT1, ATB^0,+^ and rBAT, but not ASCT1, were lowest in caecum (*P* ≤ 0.05). Expression of B^0^AT1 was higher in males vs. females (*P* ≤ 0.05). Expression of ASCT1 was higher with DL-Met and DL-HMTBA supplements compared to l-Met and control (*P* ≤ 0.05). These findings indicate that jejunum is the main intestinal segment for Na^+^-dependent l-Met and 3-OMG absorption in broilers with minor effects of dietary Met source. A sexual dimorphism for duodenal nutrient uptake and mRNA abundance of B^0^AT1 was congruent with the more efficient growth performance of male chickens known from the literature.

## Introduction

Current poultry production targets at reducing productions costs and nitrogen excretion into the environment while maintaining an effective animal performance. The key to limit nitrogen excretion is a reduction of the protein content of the diet with concurrent supplementation of deficient and (performance-)limiting amino acids (**AA**). The aim is to provide balanced quantities of individual AA at the sites of protein synthesis, which have to be further balanced with glucose availability (Lindberg, 2023). While the achievability of this aim has been studied extensively, the diverse contributing factors are still incompletely understood.

Dietary methionine (**MET**) supplements are almost regularly used in conventionally farmed poultry because Met is the first limiting AA (Vieira et al., 2004). Methionine contributes to many physiological processes including protein synthesis, feather growth, polyamine synthesis and the provision of sulfur and methyl groups (Laurino et al., 2017; Klünemann et al., 2024). Via synthesis of l-cysteine (**CYS**), Met participates in the formation of the cytoprotective molecule glutathione, thereby strengthening the intestinal barrier integrity (Wu et al., 2004). Specifically for chickens, Ruan et al. (2018) reported that Met deficiency enhanced cellular apoptosis in the small intestine of broilers, decreased the activity of antioxidative enzymes like glutathione peroxidase and increased the malondialdehyde content in their small intestine.

The major Met supplements used in conventional poultry farming are DL-Met and DL-2-hydroxy‑4-(methylthio) butanoic acid (**DL-HMTBA**). l-Met is primarily used in humans but can also be used as supplement in animal nutrition (To et al., 2021). Several studies investigated differences in efficacies of these Met sources and came to partially controversial conclusions especially about the adequacy of DL-Met versus DL-HMTBA supplements (Lemme et al., 2002; Vazquez-Anon et al., 2006; Wang et al., 2019). In a recent meta-analysis, Lemme et al. (2024) reported a relative bio-efficacy of DL-HMTBA close to 65 % relative to DL-Met in broiler chickens.

The reason for differences in bio-efficacy of different Met supplements could include different metabolic conversions or different nutrient absorption (Maenz et al., 1996; Drew et al., 2003). Interestingly, when compared to mammals, chickens exhibit nutrient absorptive capacities throughout their entire intestinal tract, including the large intestinal regions (Vinardell et al., 1986; Moreto et al., 1989). Met absorption can occur through multiple Na^+^-dependent and Na^+^-independent carrier-mediated systems. The primary pathway for l-Met absorption across the brush border membrane is seen in the Na^+^-dependent B^0^AT1 (SLC6A19) transporter that serves for low affinity absorption of a wide variety of neutral AA (*K*_m_ = 1.5 - 4.5 mM) (Broer et al., 2004). In addition to B^0^AT1, the ATB^0,+^ (SLC6A14) transporter facilitates Met absorption in a 2:1:1 ratio of Na^+^, Cl^−^, and Met molecules (*K*_m_ ∼14 µM) (Mastrototaro et al., 2016). Met absorption across the apical membrane of the chicken small intestine is further maintained through the Na^+^-independent AA exchanger b^0,+^/rBAT (SLC7A9/SLC3A1; *K*_m_ = 71 µM) (Palacin, 1994; Torras-Llort et al., 2001). Additionally, the Na^+^- and Cl^−^-dependent IMINO transporter SIT1 (SLC6A20) was reported to accept Met with much lower affinity, at least in mammals (*K*_m_ = 6.9 mM) (Nickel et al., 2009). However, data regarding its expression in the chicken small intestine is limited (Lerner et al., 1978).

After feeding different Met supplements, Zhang et al. (2017) observed enhanced expression of ATB^0,+^ and B^0^AT1 transporters in the small intestine of broilers that received DL-Met or l-Met; whereas, HMTBA supplementation had no effect. In contrast, Wang et al. (2019) stated that supplementation of HMTBA in broiler chickens resulted in lower mRNA expression of B^0^AT1 and ATB^0,+^ in the duodenum of broiler chickens. Our group reported an increased mRNA expression of ASCT2 (SLC1A5) and the functional induction of Na^+^-dependent Met absorption in the jejunum of pigs receiving DL-Met supplementation when compared with l-Met and DL-HMTBA supplementation (Romanet et al., 2021). Whether the ASC system is also regulated by dietary Met supplements in chickens is currently not known. Chickens lack the gene encoding for ASCT2, a neutral AA exchanger in the brush border membrane (Gesemann et al., 2010). Instead, broilers express ASCT1 (SLC1A4) in the apical and basolateral membrane of the small intestine (Kaminski and Wong, 2018; Miska et al., 2019; Wang et al., 2022), which belongs to the same protein family but shows a more narrow substrate range than ASCT2 (Wang et al., 2022).

As mentioned earlier, protein accretion in poultry is greatly dependent on balanced glucose supply (Azahan et al., 1989; Lindberg, 2023). In contrast to the multiple and partly redundant transporters available for intestinal AA absorption, the Na^+^-dependent absorption of glucose across the apical membrane relies primarily on the Na^+^-d-glucose linked transporter, SGLT1 (SLC5A1) (Gilbert et al., 2007). This transporter plays a pivotal role in facilitating absorption of carbohydrates to ensure energy supply (Sklan et al., 2003). In functional studies 3-O-methyl-d-glucose (**3-OMG**) is often used as non-metabolizable glucose analogue to characterize SGLT1 function (McWhorter et al., 2010).

It has already been reported that Met supplementation is known to influence glucose absorption (Giurgea et al., 1991) and previous studies revealed differences in the uptake of l-Met and mRNA abundance of Met transporters in the gastrointestinal tract of chickens and pigs receiving different Met sources (Zhang et al., 2017; Romanet et al., 2020; 2021). This study hypothesized that supplementation of different Met sources affects the absorption of l-Met and 3-OMG in duodenum (**DUO**), mid-jejunum (**JEJ**) and caecum (**CAE**). Furthermore, the study aimed to investigate the mRNA expression levels of relevant transporters for Met and glucose across the three investigated intestinal sections. Because male and female chicken may respond differently to dietary treatments based on their different growth potential, sex differences were also considered.

## Materials and methods

The animal experiments were declared to the responsible animal care and use authority, the 'Landesamt für Gesundheit und Soziales Berlin' (LaGeSo Reg. No: T 0033/19).

### Animals and diets

**Fig. 1:**
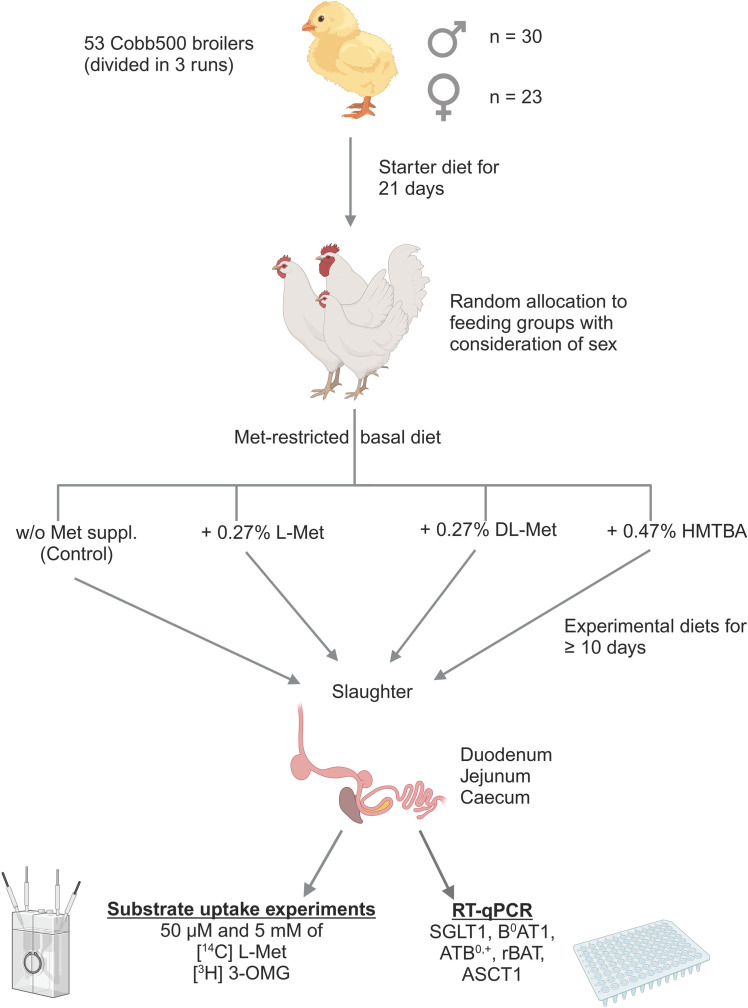
Graphical sketch of the experimental design. See text for details. ASCT1 = Neutral amino acid transporter A; ATB^0,+^ = Amino acid transporter B^0,+^; B^0^AT1 = B^0^ amino acid transporter 1; HMTBA = 2-hydroxy-4-(methylthio) butanoic acid; Met = methionine; 3-OMG = 3-O methyl-d-glucose; rBAT = heavy chain of neutral and basic amino acid transport protein; SGLT1 = Sodium-d-glucose linked transporter 1; suppl. = supplement. Created in BioRender. Aschenbach, J. (2025) https://BioRender.com/41v0ewb.

The general outline of the experimental design is visualized in Fig. 1. In three consecutive runs, a total of 53 (30 male and 23 female) Cobb500 broilers were purchased as day-old chickens from Cobb Germany Avimex GmbH (Wiedemar, Germany). Chickens were floor-housed on wood chips litter at the Institute of Animal Nutrition, Freie Universität Berlin, Germany and had *ad libitum* access to feed and water. Light was maintained from 0800 h till 1800 h with 30 Lux in compliance with the recommendations of the German Agricultural Society (DLG Ausschuss Technik in der Tierhaltung, 2018). Diets were purchased from Research Diet Services BV (NC Wijk bij Duurstede, The Netherlands) and formulated to meet or exceed the recommendations of the breeder (CobbandVantress, 2022), except for a marginal Met supply in the experimental diet of the control group as part of the dietary intervention.

Initially all animals received a starter diet for 21 days (feed ingredients and nutritional composition in Table 1, Table 2). Thereafter, male and female chickens were randomly divided into four feeding groups (3 - 5 birds per group per run), taking gender into account. One group served as control group and received a basal grower-finisher diet that was limited in digestible Met + Cys (0.49 %). For one each of the three remaining feeding groups, the basal diet was supplemented with either 0.27 % l-Met, 0.27 % DL-Met (MetAMINO, Evonik Operations GmbH, Germany) or 0.47 % DL-HMTBA (MHA-Sipernat; Evonik Nutrition *&* Care, Essen, Germany; containing 63 % liquid MHA (Rhodimet AT88; Adisseo France S.A.S, Antony, France) on 37 % Sipernate). The DL-HMTBA diet was slightly over-supplemented to account (1) for the lower bio-efficacy of DL-HMTBA (∼65 % compared to DL-Met; (Lemme et al., 2002; 2020; 2024)) and (2) for the fact that even bioefficacy-corrected feed supplementation of DL-HMTBA leads to much lower increases of plasma Met concentrations compared to dietary DL-Met supplementation (Yodseranee et al., 2012). All feeding groups were blinded to the research team until the whole data set was collected and analyzed.

**Table 1:** Feed ingredients (%, as fed basis) and calculated nutrient composition of starter and experimental diets.

	Starter diet	Grower-finisher diet			
Ingredients		DL-Met	L-Met	DL-HMTBA	Control
Corn	55.40	61.17	61.17	61.17	61.17
Soybean meal, 48 % CP	36.65	29.59	29.59	29.59	29.59
Soybean oil	3.45	4.65	4.65	4.65	4.65
Dicalcium phosphate	1.92	1.76	1.76	1.76	1.76
Limestone (CaCO_3_)	0.91	0.83	0.83	0.83	0.83
Premix Blank Poultry^1^	0.50	0.50	0.50	0.50	0.50
Salt (NaCl)	0.23	0.23	0.23	0.23	0.23
Sodium bicarbonate	0.22	0.22	0.22	0.22	0.22
L-Lysine HCL^2^	0.20	0.15	0.15	0.15	0.15
L-Threonine^2^	0.08	0.07	0.07	0.07	0.07
L-Valine^2^	0.07	0.04	0.04	0.04	0.04
Choline Chloride 60 %	0.03	0.04	0.04	0.04	0.04
Filler^3^	0.00	0.47	0.47	0.000	0.74
DL-Methionine^2^	0.34	0.27	0.00	0.00	0.00
L-Methionine^2^	0.00	0.00	0.27	0.00	0.00
MHA-SIPERNAT^2^^,^^4^	0.00	0.00	0.00	0.74	0.00

Calculated nutrient composition					

Dry matter	87.7	87.8	87.8	87.8	87.8
Crude protein	22.2	18.9	18.9	18.9	18.9
Crude fiber	2.94	2.77	2.77	2.77	2.77
Crude fat	6.15	7.40	7.40	7.40	7.40
AMEn (MJ/kg)	12.6	13.1	13.1	13.1	13.1
SID^5^ Lysine	1.22	1.01	1.01	1.01	1.01
SID Methionine	0.62	0.52	0.52	0.52	0.25
SID Methionine + Cysteine	0.89	0.76	0.76	0.76	0.49
SID Threonine	0.77	0.66	0.66	0.66	0.66
SID Tryptophan	0.24	0.20	0.20	0.20	0.20
SID Valine	0.96	0.81	0.81	0.81	0.81
SID Glycine + Serine	1.71	1.48	1.48	1.48	1.48
SID Arginine	1.34	1.13	1.13	1.13	1.13
SID Isoleucine	0.83	0.71	0.71	0.71	0.71
SID Leucine	1.58	1.40	1.40	1.40	1.40
SID Phenylalanine	0.96	0.83	0.83	0.83	0.83
SID Phenylalanine + Tyrosine	1.75	1.51	1.51	1.51	1.51
SID Histidine	0.51	0.44	0.44	0.44	0.44
Calcium	0.96	0.87	0.87	0.87	0.87
Phosphorus	0.72	0.65	0.65	0.65	0.65
Sodium	0.16	0.16	0.16	0.16	0.16
Chloride	0.22	0.21	0.21	0.21	0.21

1 Containing per kilogram premix: vitamin A (retinyl acetate), 2,000,000 IU; vitamin D3 (cholecalciferol), 500,000 IU; vitamin E (DL-a-tocopherol), 10 g; vitamin K3 (menadione), 300 mg; vitamin B1 (thiamin), 400 mg; vitamin B2 (riboflavin), 1.5 g; vitamin B6 (pyridoxin-HCL), 700 mg; vitamin B12 (cyanocobalamin), 4 mg; niacin, 7 g; D-pantothenic acid, 2.4 g; choline chloride, 92 g; folic acid, 200 mg; biotin, 40 mg; iron, 16 g; copper, 2.4 g; manganese, 17 g; zinc, 12 g; iodate, 160 mg; selenium, 30 mg.

2 Evonik Operations GmbH, Essen, Germany.

3 Diamol® DI 100 G (Damolin A/S, Fur, Denmark) containing per kg premix 690 g SiO_2_, 70 g Fe_2_O_3_, 90 g Al_2_O_3_, 10 g TiO_2_, 10 g CaO, 20 g K_2_O + Na_2_O, 10 g MgO.

4 Containing 63 % liquid DL-2-hydroxy-4-(methylthio) butanoic acid (DL-HMTBA; Rhodimet AT88®, Adisseo France S.A.S, Antony, France) on 37 % Sipernate).

5 SID = standardized ileal digestibility.

**Table 2:** Analyzed values of starter and experimental diets (%, as fed basis).

	Starter diet	Grower-finisher diet			
Nutrient composition		DL-Met	L-Met	DL-HMTBA	Control
Dry Matter	89.0	88.6	88.6	88.3	88.6
Crude protein	23.3	19.9	19.9	19.6	19.4
Crude fiber	2.7	2.7	2.6	2.6	2.7
Ether extract	6.5	6.6	6.7	7.0	6.9
Ash	5.6	5.3	5.2	4.9	5.3
Neutral detergent fiber	8.5	9.1	8.6	9.1	8.8
Acid detergent fiber	3.6	3.6	3.5	3.4	3.6
Starch	37.4	40.5	41.3	41.2	41.4
Sugar	4.7	4.0	4.1	4.2	3.8
Methionine	0.66	0.54	0.53	0.28	0.27
Cysteine	0.34	0.30	0.30	0.30	0.29
Methionine + Cysteine^1^	1.00	0.84	0.83	0.58	0.56
Lysine	1.41	1.15	1.17	1.16	1.14
Threonine	0.94	0.79	0.80	0.80	0.78
Arginine	1.5	1.25	1.27	1.27	1.24
Isoleucine	0.97	0.81	0.82	0.82	0.80
Leucine	1.82	1.60	1.63	1.62	1.58
Valine	1.14	0.94	0.94	0.94	0.93
Histidine	0.57	0.49	0.49	0.49	0.48
Phenylalanine	1.14	0.98	0.97	0.97	0.95
Glycine	0.92	0.78	0.79	0.79	0.77
Serine	1.08	0.92	0.94	0.93	0.91
Proline	1.21	1.10	1.10	1.10	1.07
Alanine	1.05	0.94	0.94	0.94	0.92
Asparagine	2.33	1.94	1.96	1.95	1.91
Glutamic acid	3.89	3.35	3.37	3.36	3.28
Ammonia	0.45	0.39	0.39	0.39	0.38
Supplemented					
Methionine	0.34	0.26	0.27	<0.10	<0.10
DL-HMTBA^2^				0.48	

1 Not including DL-2-hydroxy-4-(methylthio) butanoic acid.

2 DL-HMTBA = DL-2-hydroxy-4-(methylthio) butanoic acid.

### Feed analysis

Feed ingredients were analyzed by Evonik (Hanau-Wolfgang, Germany). Near-infrared spectroscopy (NIRS) was used to determine crude fiber, ether extract, ash, neutral detergent fiber, acid detergent fiber, starch, sugar and dry matter contents according to ISO 12099:2017 (ISO 12099, 2017). The method of Llames and Fontaine (1994) was applied for chemical analysis of crude protein and AA contents, using Leco FP-2000 Nitrogen Analyzer (Leco Corporation, St. Joseph, MI, USA) for the determination of nitrogen.

### Tissue preparation and mounting

After a minimum of ten days on the grower-finisher diet, two chickens per day were stunned and killed by exsanguination with a total of 53 animals used throughout the study. Selection for slaughter was random within groups; however, selection for slaughter was rotated between the groups to achieve the same mean time on the grower-finisher diet for each group over the course of the whole trial.

Tissue harvest and incubation followed standard procedures (Awad et al., 2004; 2007) with adaptations derived from recent optimization trials that have been partly published (Romanet et al., 2025). Immediately after slaughter, an incision was made along the caudal rim of the sternum. Tissue sections of DUO, JEJ and CAE were obtained and immediately rinsed in ice-cold buffered transport solution without (DUO and JEJ) or with short chain fatty acids (CAE). The composition of all solutions is listed in Table 3. Solutions were pre-gassed with carbogen (95 % O_2_ and 5 % CO_2_).

**Table 3:** Composition of mucosal and serosal bathing solutions (mmol·L^-1^) 1.

	Duodenum and jejunum			Caecum		
Composition	Serosal 2	Mucosal + Na^+^	Mucosal Ø Na	Serosal 2	Mucosal + Na^+^	Mucosal Ø Na
NaCl	120.00	120.00		120.00	80.00	
NMDGCl 3			125.40			85.40
NaHCO_3_	25.00	25.00		25.00	25.00	
Choline-HCO_3_			25.00			25.00
KCl	5.40	5.40		5.40	5.40	
NaH_2_PO_4_	0.60	0.60		0.60	0.60	
Na_2_HPO_4_	2.40	2.40		2.40	2.40	
KH_2_PO_4_			0.60			0.60
K_2_HPO_4_			2.40			2.40
HEPES	10.00	10.00	10.00	10.00	10.00	10.00
Glucose	20.00			20.00		
Mannitol	5.00	25.00	25.00	5.00	25.00	25.00
Na-acetate					25.00	
Na-propionate					10.00	
Na-butyrate					5.00	
NMDG (free base)						40.00
Acetic acid						25.00
Propionic acid						10.00
Butyric acid						5.00
MgCl_2_	1.20	1.20	1.20	1.20	1.20	1.20
CaCl_2_	3.00	3.00	3.00	1.50	1.50	1.50
Amino acid mix 4	1.4	no	no	1.4	no	no

1 All bathing solutions were adjusted to pH 7.4 ± 0.03, 320 ± 10 mosmol·L^−1^.

2 Glucagon-like peptide-1 (GLP-1) was added to the serosal side of the Ussing chamber (final concentration per chamber, 25 nmol·L^−1^) to support tissue vitality. Bovine serum albumin (final concentration per chamber, 100 mg·L^−1^) was used to prevent unspecific binding to the equipment and Sab Simplex (final concentration per chamber: 6.92 mg·L^−1^) was added to avoid excessive foaming. Serosal solutions also served as transport solutions.

3 NMDG = N-methyl-d-glucamine.

4 Composition of the amino acid mix can be found in (Romanet et al., 2020).

The serosal layer was mechanically stripped off the JEJ. Stripping of the DUO and CAE could not be reliably performed without damaging the epithelial layer; therefore, experiments for DUO and CAE were performed with whole-mount tissues. Tissue samples were transported to the laboratory in ice cold transport solution (Table 3) and were continuously gassed with carbogen. Epithelia were cut into squares (∼ 2 cm^2^) and mounted in conventional Ussing chambers (solution-exposed area, 0.95 cm^2^) containing rings of silicone rubber on each side of the tissue to reduce tissue damage resulting from contusion.

### Ussing chamber experiments

Tissue samples were incubated in 12 mL of buffered solution on both the mucosal and serosal sides. The bathing solutions were gassed with carbogen over the whole experimental duration and held constant at 38°C by a thermostat. Composition of the bathing solutions differed for mucosal and serosal sides and for tissues (Table 3). Experiments were performed in the presence and absence of Na^+^ with N-methyl-d-glucamine (**NMDG^+^**) and choline serving as sodium replacements.

Initially, all chambers were filled with Na^+^-containing solution on the mucosal side. After a short equilibration time (∼5 min) under open circuit conditions, Ussing chambers were short-circuited, i.e., *PD*_t_ was clamped to 0 mV. After another ∼5 min under short circuit conditions, all chambers were paired according to their *G*_t_ values to generate uptake pairs with highly similar functionality. High and low-*G*_t_ chamber pairs were alternated between the three consecutive runs to achieve similar mean *G*_t_ values for the used uptake substrates and concentrations.

The uptake protocol for each chamber pair started with exchanging the buffered solution on the mucosal side of the Ussing chamber to new Na^+^-containing or Na^+^-free solution. After 3 min of equilibration, a radioisotope mix consisting of 74 kBq [^3^H]−3-OMG and 74 kBq [^14^C]-l-Met was added to the mucosal side. The final concentration of l-Met and 3-OMG was adjusted to 50 µM or 5 mM by the addition of an appropriate amount of unlabeled l-Met and 3-OMG to the radioisotope mix. At ∼20 s after adding radioactivity, two samples (2 × 100 µL) were taken from the solution on the mucosal side. Exactly 1 min after adding the radioisotope mix, bathing solutions on both sides of the tissue were released and the tissue was rinsed three times with 12 mL ice-cold Na^+^-free solution to wash off all adhering radioactivity and to stop transport processes. Afterwards, the tissue was removed from the chamber and tissue samples (0.42 cm^2^) were punched out of the middle of the solution-exposed area. Punched-out samples were placed in vials containing 2.5 mL lysis solution (0.2 M NaOH and 0.25 % SDS) and were vortexed for 30 s. Thereafter, vials were placed on a rotating agitator for 4 min and vortexed again for 30 s.

The remaining tissue was removed from the vials, which were then centrifuged at 5000 g at 4°C for 20 min. Afterwards two samples (2 × 600 µL) were taken from the lysate. Volume in hot samples was adjusted to 600 µL with 500 µL lysis solution. In the following step, 3 mL of scintillation fluid (Aquasafe 300 Plus, Zinsser Analytic GmbH, Frankfurt am Main, Germany) was added to all samples. Radioactivity was counted using a liquid scintillation β-counter (TRI-CARB 4910TR, Perkin-Elmer, Rodgau, Germany). The disintegrations per minute (**DPM**) were counted with detection windows of 1 to 18.6 keV and 4 to 156 keV for ^3^H and ^14^C, respectively.

Uptakes were calculated in nmol·cm^−2^·min^−1^ using the following equation:

$$U=\frac{V_{t}\times C_{n}\times \frac{dpm_{n}\times V_{l}}{V_{c}}}{\frac{dpm_{h}\times V_{t}}{V_{h}}\times A}$$

where C_n_, AA concentration in the mucosal solution [M]; V_t_, total solution volume of mucosal solution [L]; V_l_, volume used for lysis [L]; V_c_, volume of counted aliquot of lysis solution [L]; V_h_, volume of mucosal solution sample [L]; dpm_n_, radioactivity in the counted aliquot of lysis solution at AA concentration Cn [DPM]; dpm_h_, radioactivity in mucosal solution sample [DPM] and A, surface area of lysed tissue [cm²].

### Reverse-transcription quantitative PCR (RT-qPCR)

Epithelial samples of cleaned DUO, JEJ and CAE were obtained right after slaughter by perpendicular cuts with a scissor and stored in RNA*later* (Sigma-Aldrich, St. Louis, USA) at -20°C. Total RNA was extracted using a commercial kit (Nucleospin RNA purification, Macherey & Nagel, Düren, Germany) following the manufacturer's instructions. For quality and quantity control, the concentration and purity ratios 260/230 nm and 260/280 nm of each RNA sample were determined by an IMPLEN NanoPhotometer P330 (Implen GmbH, Munich, Germany). RNA integrity was checked by a lab-on-a-chip electrophoretic separation (Agilent RNA 6000 Nano Kit, Agilent Technologies, Waldbronn, Germany). All samples that were used for cDNA synthesis showed an RNA integrity number (RIN) ≥ 6.

An iScript cDNA Synthesis kit (Bio-Rad, Hercules, CA, USA) and Mastercycler nexus (Eppendorf SE, Hamburg, Germany) were used to perform reverse transcription of 1000 ng·µL^−1^ RNA per sample. The following PCR thermocycler protocol was applied according to the test kit instructions: priming at 25°C for 5 min, reverse transcription at 46°C for 20 min and denaturation of cDNA at 95°C for 1 min. To check for possible contamination with genomic DNA, minus-RT samples were generated for each RNA sample without adding the RT enzyme mix. All cDNA samples were diluted 1:10 with ddH_2_O.

Differences in relative mRNA expression of the genes for Met transporters *B^0^AT1, ATB^0,+^, ASCT1, rBAT* as heavy chain of b^0,+^AT/rBAT and glucose transporter *SGLT1* were examined in the qPCR step. Exon-spanning primers were designed using Primer3Plus (Michelstadt, Germany) and synthesized by Eurofins Genomics (Ebersberg, Germany, see primer sequences in Table 4). Primer pairs for system IMINO could not be established. A 40-cycle two-step method (95°C for 15 s and 58°C for 1 min) was applied using a thermocycler (ViiA7, Applied Biosystems/Life Technologies; Foster City, CA, USA) and iQ™SYBR Green Supermix Kit (Bio-Rad, Hercules, CA, USA). All experiments were performed in 384-well plates with triplets of each sample using 5 µL of cDNA and 15 µL of a master mix with added SYBRGreen and the respective primers. An inter-run calibrator (IRC) comprising an aliquot of all cDNA samples was used to calibrate among the plates. Nuclease-free water served as negative control sample. To check for genomic DNA contamination, an RT-qPCR was conducted on a pooled sample of all minus-RT samples, which was also run on each plate. Glycerinaldehyde-3-phosphate-dehydrogenase (***GAPDH***) and TATA-box binding protein (***TBP***) served as housekeeping genes for normalization. Stable expression was checked for both genes using geNorm (Vandesompele et al., 2002).

**Table 4:** Primer sequences for SGLT1, Met transporters and reference genes.

Gene	SLC	Accession number	Primer	Sequence 5′−3′
*SGLT1*	SLC5A1	NM_001397792.1	Forward	CCTTCCAACTGTCCGTTCAT
			Reverse	ATCGGGTTTCTCCTCCTCAT
*B^0^AT1*	SLC6A19	XM_419056.8	Forward	CTTCTGCCTGGGTTTGTCAT
			Reverse	AATGGAGCCAGCAAAACTGT
*ATB^0,+^*	SLC6A14	XM_015278436.4	Forward	GTGGAGGATGTGCTGGTTTT
			Reverse	ACGATGGGAATCCAGATGAC
*ASCT1*	SLC1A4	XM_046939644.1	Forward	GCGACGTTTCCTCTTCAGAC
			Reverse	GCGACGTTTCCTCTTCAGAC
*rBAT*	SLC3A1	XM_004935370.5	Forward	CAAACACTGCTCGGATTTCA
			Reverse	TGCATAGGGGATTTCTCTGG
*GAPDH*		NM_204305.2	Forward	CCAACCCCCAATGTCTCTGT
			Reverse	CATTCAGTGCAATGCCAGCA
*TBP*		NM_205103.2	Forward	AGGATTGGTACTCACACACCA
			Reverse	GGTTGTCTTCCGGAACCCCTT

SGLT1 = Sodium-d-glucose linked transporter 1; B^0^AT1 = B^0^ amino acid transporter 1; ATB^0,+^ = Amino acid transporter B^0,+^; ASCT1 = Neutral amino acid transporter A; rBAT = heavy chain of neutral and basic amino acid transport protein; GAPDH = Glycerinaldehyde-3-phosphate dehydrogenase; TBP = TATA box binding protein.

### Statistical analysis

The individual chicken was considered as experimental unit. Statistical analysis was performed using Sigma Plot 15.0 (Systat Software GmbH, Erkrath, Germany). Data of uptake measurements were analyzed using sets of three-way ANOVA comparing the factors tissue (DUO, JEJ, CAE), sodium (Na^+^, NMDG^+^), diet (L-Met, DL-Met, DL-HMTBA, Con) and sex (male, female) in all possible combinations of factors (Supplementary Table 1). For post-hoc pairwise comparisons, Student-Newman-Keuls' test was used. Because of missing normal distribution, data was square-rooted before analysis and back-transformed thereafter.

For analyzing RT-qPCR data, the 2^−∆∆Ct^ method was applied. Two reference genes served for normalization and a pool consisting of all cDNA samples was used for calibration. Significant outliers of sample triplets were removed using a Grubb's test. Statistical analysis was performed using a three-way ANOVA comparing the factors tissue, diet and sex. For post-hoc pairwise comparison, a Student-Newman-Keuls' test was used.

All results are given as least square means ± 95 % CI. Differences of *P* ≤ 0.05 were considered significant and 0.05 < *P* ≤ 0.10 was considered a trend. The number of experimental animals used is given as n.

## Results

### Apical uptakes of 3-OMG

**Fig. 2:**
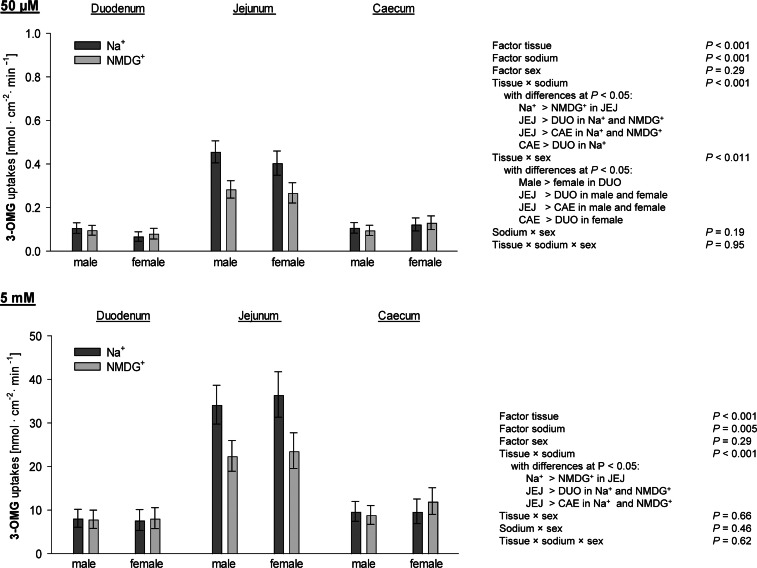
Uptakes of 3-O methyl-d-glucose (3-OMG) at a final concentration of 50 µM and 5 mM. Columns represent least square means ± CI from *n* = 19 - 30 chickens. Three-way ANOVA and post hoc Student-Newman-Keuls' test were used to compare the factors tissue, sodium, sex and their two- and three-way interactions. DUO = duodenum; JEJ = jejunum; CAE = caecum; NMDG = N-methyl-d-glucamine.

Statistical analysis of 3-OMG uptakes did not show any effect for the factor diet at both tested concentrations (Supplementary Table 1). Therefore, the factor diet was excluded from the model and results are solely presented for the three-way ANOVA comparing the factors tissue, sodium, sex and their interactions (Fig. 2).

Uptakes of 3-OMG at a final mucosal concentration of 50 µM were affected by the factors tissue and sodium (*P* < 0.001, each) with significant tissue × sodium (*P* < 0.001) and tissue × sex interactions (*P* = 0.011). The interaction tissue × sodium revealed, on the one hand, that JEJ had higher 3-OMG uptakes than DUO and CAE both in the presence and in the absence of mucosal Na^+^ (*P* < 0.001), with CAE showing (*P* = 0.024; presence of Na^+^) or tending to show (*P* = 0.069; absence of Na^+^) higher uptakes than DUO. On the other hand, the post-hoc comparison of the tissue × sodium interaction revealed a significant decrease of 3-OMG uptakes due to replacement of Na^+^ by NMDG^+^ in only JEJ (*P* < 0.001) but not in DUO and CAE. With regard to the tissue × sex interaction, female chickens showed lower uptakes than male chickens in DUO (*P* = 0.019), whereas female chickens tended to show higher 3-OMG uptakes in CAE (*P* = 0.074) at 50 µM final concentration. This was additionally linked to the finding that 3-OMG uptakes were not different between DUO and CAE in male chickens, whereas, all other combinations of tissues showed different uptakes across sexes (*P* < 0.001) with JEJ being highest and DUO being lowest.

At a final mucosal concentration of 5 mM, 3-OMG uptakes were also affected by the factors tissue (*P* < 0.001) and sodium (*P* = 0.005) and by a tissue × sodium interaction (*P* < 0.001). Similar to 50 µM, post hoc testing of the tissue × sodium interaction revealed that uptakes of 3-OMG were higher in JEJ than in DUO and CAE, both in the presence and absence of Na^+^ (*P* < 0.001), with Na^+^-dependence of 3-OMG uptakes being evident in JEJ only (*P* < 0.001). Uptakes of 3-OMG at 5 mM final mucosal concentration were not affected by the factor sex or any interaction with the factor sex.

### Apical uptakes of l-methionine

**Fig. 3:**
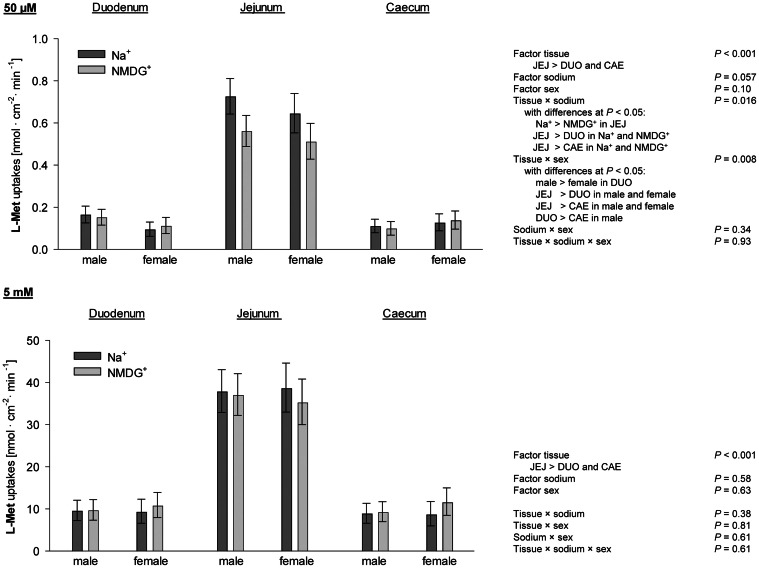
Uptakes of l-methionine (L-Met) at a final concentration of 50 µM and 5 mM. Columns represent least square means ± CI from *n* = 19 - 30 chickens. Three-way ANOVA and post hoc Student-Newman-Keuls' test were used to compare the factors sodium, tissue, sex and their two- and three-way interactions. DUO = duodenum; JEJ = jejunum; CAE = caecum; NMDG = N-methyl-d-glucamine.

Because the factor diet had no influence on apical uptakes of l-Met at a concentration of 50 µM l-Met in any three-way combinations tested (Supplementary Table 1), uptakes at 50 µM l-Met were evaluated only for the factors tissue, sodium and sex (Fig. 3). Uptakes were affected by the factor tissue (*P* < 0.001) and, as a trend, sodium (*P* = 0.057), with significant tissue × sodium (*P* = 0.016) and tissue × sex interaction (*P* = 0.008). The tissue × sodium interaction was based on higher uptakes in JEJ compared to DUO and CAE in the presence and absence of Na^+^ (*P* < 0.001) with no difference between DUO and CAE (*P* > 0.05). Furthermore, uptakes of l-Met were strongly decreased by the absence of Na^+^ in JEJ only (*P* < 0.001), whereas uptakes in DUO and CAE showed no differences in the presence vs. absence of mucosal Na^+^. Regarding the tissue × sex interaction, post hoc comparison revealed that female chickens showed lower uptakes of l-Met in DUO (*P* = 0.004). The latter was linked to the additional finding that l-Met uptakes were not different between DUO and CAE in female chickens, whereas tests for all other tissue combinations showed differences across sexes (*P* ≤ 0.05) with JEJ being highest and CAE being lowest.

At a concentration of 5 mM, the three-way comparison for the factors tissue, sodium and sex showed that l-Met uptakes were affected solely by the factor tissue (*P* < 0.001). Jejunum showed higher uptakes than DUO and CAE (*P* ≤ 0.05) with the latter two being not different from each other (*P* > 0.05). An effect of sodium on l-Met uptakes could not be observed in all three intestinal segments at 5 mM l-Met (*P* > 0.05).

**Fig. 4:**
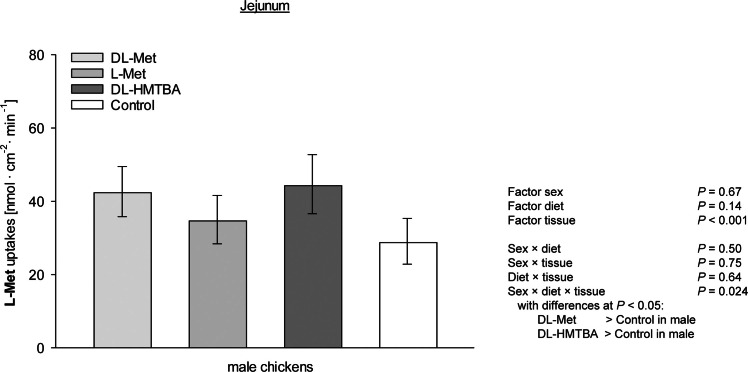
Three-way interaction of uptakes of l-methionine (L-Met) at a final concentration of 5 mM for the factors tissue, diet and sex as further isolated by post hoc Student-Newman-Keuls' test in jejunum of male chickens. Columns represent least square means ± CI from *n* = 6 - 8 chickens. Data for female chickens in jejunum and for male and female chickens in duodenum and caecum are not shown because they did not show diet-dependent differences (*P* > 0.05). HMTBA = 2-hydroxy-4-(methylthio) butanoic acid.

The factor tissue was also significantly influenced in the three-way ANOVA comparing the factors tissue, sex and diet (*P* < 0.001). However, this comparison additionally revealed a significant tissue × diet × sex interaction (*P* = 0.024; Fig. 4). The latter interaction effect was restricted to the JEJ where male chickens showed higher uptakes after receiving DL-Met or DL-HMTBA compared to CON (*P* ≤ 0.05).

### RT-qPCR

**Fig. 5:**
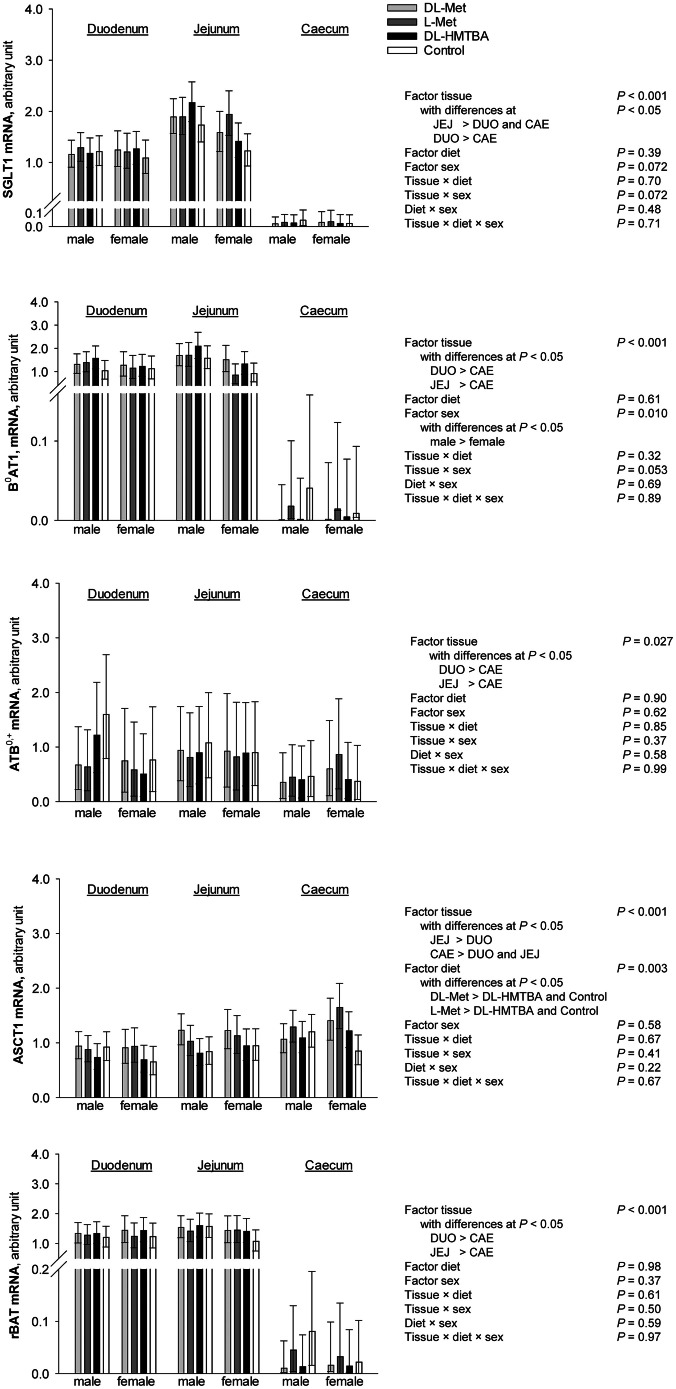
mRNA expression of selected glucose and methionine transporters in duodenum, jejunum and caecum using RT-qPCR from *n* = 5 - 8 chickens. Data was compared using three-way ANOVA (factors tissue, diet and sex) and post hoc Student-Newman-Keuls' test. DUO = duodenum; JEJ = jejunum; CAE = caecum; Met = methionine; HMTBA = 2-hydroxy-4-(methylthio) butanoic acid; SGLT1= Sodium-d-glucose linked transporter 1; B^0^AT1 = B^0^ amino acid transporter 1; ATB^0,+^ = Amino acid transporter B^0,+^; ASCT1 = Neutral amino acid transporter A; rBAT = heavy chain of neutral and basic amino acid transport protein.

Results of RT-qPCR analysis of transporter gene abundance is presented in Fig. 5. When comparing the factors tissue, diet and sex, all transporter mRNA were affected by the factor tissue (ATB^0,+^, *P* = 0.027; all other transporters, *P* < 0.001). Upon post-hoc Student-Newman-Keuls' test, expression levels of SGLT1, B^0^AT1, ATB^0,+^ and rBAT showed a similar pattern with lower values in CAE compared to DUO and JEJ (*P* ≤ 0.05). The mRNA abundance in DUO and JEJ did not differ significantly for B^0^AT1, ATB^0,+^ and rBAT (*P* > 0.05); however, it was greater in JEJ compared to DUO for SGLT1 (*P* ≤ 0.05). Unlike the mentioned transporters, the mRNA abundance of ASCT1 was highest in CAE. It then decreased in JEJ and further in DUO (*P* ≤ 0.05).

A significant effect of the factor diet was observed for the mRNA abundance of ASCT1 only (*P* = 0.003). Post-hoc comparison revealed that chicken receiving DL-Met or l-Met supplements exhibited higher ASCT1 expression levels in comparison to DL-HMTBA-receiving chickens and the control group (*P* ≤ 0.05). Expression levels between DL-Met and l-Met groups as well as between DL-HMTBA and control groups did not differ significantly (*P* > 0.05).

With regard to the factor sex, only B^0^AT1 showed significantly different mRNA abundance levels (*P* = 0.010) that were higher in male vs. female chickens. A trend for higher mRNA abundance levels in male chickens was also observed for SGLT1 (*P* = 0.072). A tissue × sex interaction was indicated for both transporters (B^0^AT1, *P* = 0.053; SGLT1, *P* = 0.072) but did not reach statistical significance. All other transporters were not affected by the factor sex (*P* > 0.05).

## Discussion

Previous experiments in chickens indicated increased mRNA expression of selected AA transporters in their small intestine when supplemented with dietary DL-Met and l-Met but not DL-HMTBA (Zhang et al., 2017). Our group further showed functional changes in l-Met uptake when supplementing DL-Met but not l-Met and DL-HMTBA to young pigs (Romanet et al., 2020; 2021). Triggered by these findings, the present investigation was undertaken to assess the impact of l-Met, DL-Met and DL-HMTBA supplementation on the absorptive function for l-Met in three distinct intestinal segments. In parallel, the absorptive capacity for glucose, the mRNA abundance of critical Met and glucose transporters and possible sex differences were studied.

In contrast to our initial hypothesis, we observed no main effects of dietary supplements on the uptake of L-Met and the glucose analogue, 3-OMG. This applied for the comparison of Met- and HMTBA-supplemented groups among each other, as well as to a non-supplemented control group with limiting Met supply, both at low and high substrate concentrations. The only difference was a three-way interaction of tissue × diet × sex where male chickens of the non-supplemented control group had lower uptakes of l-Met in JEJ at the high concentration of 5 mM compared to DL-Met and DL-HMTBA-supplemented chickens. The underlying mechanism of this finding remains unclear but proceeding from the fact that uptake of l-Met was not influenced by the presence of Na^+^ at 5 mM concentration, this finding could indicate a lower functional activity of Na^+^-independent l-Met transporters in male control chickens. The Na^+^-independent absorption of l-Met is mediated through rBAT (Torras-Llort et al., 2001). The finding that the mRNA expression of rBAT did not mirror the tissue × diet × sex interaction seen for l-Met uptake is not in contrast to the proposal of a different functional activity of rBAT because it was shown previously for, e.g., B^0^AT1 in rats that AA transporters are predominantly regulated at posttranscriptional levels (Jando et al., 2017). An alternative explanation could be a lower absorptive area (i.e., shorter villi) in male control chickens compared to male DL-Met- and DL-HMTBA-supplemented chickens. Both Met sources were already shown to have beneficial effects on small intestinal morphology, which could enhance AA absorption (Zhang et al., 2023). Irrespective of the precise cause, this observed sex difference could have the practical implication that especially male chickens could benefit from the supply of either DL-Met or DL-HMTBA as dietary Met source.

The JEJ is a main intestinal segment of nutrient absorption in chickens (Mitchell et al., 1981, 2008; Wang et al., 2015). Our study confirmed this by showing that l-Met and 3-OMG uptakes were highest in JEJ. Furthermore, most of the investigated AA and glucose transporters showed highest mRNA abundance in JEJ on gene abundance levels. The mRNA abundance data suggest that Na^+^-dependent transporters in JEJ have a main role in the absorption of l-Met and glucose across the brush border membrane. Since the presence of Na^+^ led to increased uptake of 3-OMG in our research, it can be assumed that the Na^+^-glucose co-transporter SGLT1 was responsible for facilitating 3-OMG absorption across the brush border membrane. Furthermore, our study revealed that mRNA levels of SGLT1 were most prominent in JEJ, aligning with similar observations made in turkeys (Weintraut et al., 2016).

In contrast to glucose absorption, Met absorption occurs through several Na^+^-dependent and Na^+^-independent transporter systems. In the present study, the Na^+^ dependence of jejunal l-Met uptake disappeared at a mucosal concentration of 5 mM, which is a physiologically relevant concentration in Met-supplemented chickens (Mitchell et al., 2008). This may point to a possible saturation of Na^+^-dependent transporters. Such saturation is expectable if the *K*_m_ value(s) of the involved transporter(s) are markedly lower than 5 mM, which is the case for most Met transporters (Mastrototaro et al., 2016).

At the low concentration of 50 µM, apical l-Met uptake was higher in the presence vs. absence of Na^+^ in JEJ. Literature data suggests that this Na^+^-dependent portion of uptake is supposedly carried by B^0^AT and/or ATB^0,+^(Miska et al., 2019). Partially aligning with this, the abundances of the Na^+^-dependent B^0^AT1 and ATB^0,+^ were higher in JEJ (and DUO) than CAE in our study. Previously, Gilbert et al. (2007) showed that mRNA abundance of B^0^AT1 was even higher in the ileum than in DUO and JEJ of broilers, a segment that was not investigated in the present study. Commonly, this qualifies B^0^AT1 and ATB^0,+^ as transporters with predominant small intestinal location. Coherent with the uptake data for l-Met, expression levels of neither B^0^AT1 nor ATB^0,+^ were affected by the factor diet. The latter partly complies with the studies of Zhang et al. (2017) and Klünemann et al. (2024) where effects of Met supplementation on the mRNA expression of AA transporters were prominent during the starter phase but not obvious at the end of the finisher phase. As such, it would be very intriguing to apply the design of the present study to chickens at an earlier age (i.e., starter phase) in future experiments.

The only mRNA that responded to dietary treatment was that of ASCT1. It was increased by l-Met and DL-Met supplementation in the present study when compared to DL-HMTBA supplementation and the control group. ASCT1 mRNA was included in the analysis because the mRNA abundance of ASCT2 was increased in a previous study in pigs by DL-Met supplementation, which coincided with an increase in Na^+^-dependent l-Met fluxes (Romanet et al., 2020; 2021). Because chickens lack the gene encoding for ASCT2 (Gesemann et al., 2010), it was of interest whether this related transporter can be similarly regulated by dietary Met supplementation, even though the transporter does likely not accept Met (Wang et al., 2022). The fact that ASCT1 mRNA was, indeed, regulated by DL-Met and l-Met in the present study supports the previous observation that AA transporters may be induced by substances for which the transporter itself has no affinity (Stein et al., 1987).

The present study additionally showed that ASCT1 mRNA abundance was highest in CAE. This would support a concept of relevant AA absorption across caecal intestinal epithelial cells. Unlike mammals, chickens were proposed to exhibit transporter-mediated nutrient absorption throughout the entire intestinal tract, including the caeca (Vinardell et al., 1986; Moreto et al., 1989). However, other studies concluded that carrier-mediated Met absorption in CAE diminishes rapidly within the first week of life (Lerner et al., 1975). Similarly, one of our previous studies found no phlorizin-inhibitable portion of apical glucose uptake in the CAE of broiler chickens at four weeks of age (Awad et al., 2015); and the present study could also not identify a clear Na^+^-dependence of 3-OMG uptake in chicken CAE. This suggests a rather negligible role of SGLT1 for glucose absorption in CAE of finisher chickens and fits with the extremely low mRNA expression levels of SGLT1 observed in this intestinal segment.

A peculiar finding was that no Na^+^-dependent uptakes of l-Met and 3-OMG could be observed in DUO. Moreover, 3-OMG uptakes in DUO were even lower than uptakes in CAE at 50 µM concentration. This did not fit to the high mRNA expression of SGLT1 and several AA transporters measured in this segment and appeared in contrast to the well-established role of DUO in nutrient absorption. Rapid absorption of glucose had been shown earlier in the DUO of broiler chickens (Riesenfeld et al., 1980); and decreasing concentration profiles of luminal l-Met suggest the same for duodenal l-Met-absorption (Mitchell et al., 2008). Nonetheless, these apparently controversial findings fit earlier observations. Amat et al. (1999) observed negligible currents (as an indication for Na^+^-dependent glucose uptake) after adding luminal glucose to duodenal epithelia ex vivo and Riesenfeld et al. (1980) observed that glucose absorption in the small intestine of chickens was apparently not saturable in situ. The latter authors suggested that this may point either to absorption by passive (i.e. paracellular) diffusion or to absorption via glucose carrier systems that are not saturated at high physiological glucose concentrations.

As regards sex effects, male chickens showed higher uptakes of l-Met and 3-OMG at 50 µM final concentration in DUO, which could plausibly support a better growth performance of male chickens due to a better ability to absorb nutrients already in the proximal intestinal segments. Furthermore, quantitative RT-PCR revealed a higher expression of B^0^AT1 in male chickens across intestinal segments, potentially supporting the better growth performance of male chickens known from the literature (Lopez et al., 2011; Benyi et al., 2015). Expression levels of SGLT1 only trended towards higher expression in male chickens in the present study. Kaminski and Wong (2018) reported a sexual dimorphism for SGLT1 at the day of hatch in chicken and Weintraut et al. (2016) reported that even adult male turkeys exhibit higher mRNA abundance of SGLT1 than female turkeys. Nonetheless, it has to be emphasized again that correlation between mRNA expression and corresponding transporter function may be little. Several other factors like greater absorptive area (Miles et al., 2006), broiler breed, protein synthesis mechanisms or circulating hormone levels can contribute to the sexual dimorphism and differential growth of male and female chickens (England et al., 2023).

In conclusion the present study could not reveal a major impact of different dietary Met sources on the transport of 3-OMG and l-Met, as well as on the mRNA expression of their transporters in finisher chickens. Retrospectively, this appears coherent with literature findings that intestinal nutrient transport is sensitive to Met restriction and the choice of dietary Met source predominantly in the starter phase. The study further revealed that JEJ is the major site of transporter-mediated 3-OMG and l-Met absorption in broiler chickens with significant involvement of Na^+^-dependent nutrient transporters. The mRNA expression of those transporters showed also very high mRNA expression values in DUO where the present study identified rather low substrate uptakes with no Na^+^-dependent component. The reasons behind this finding necessitate clarification in future experiments. As signs of sexual dimorphism, male chickens exhibited higher substrate uptakes at 50 µM 3-OMG and l-Met in DUO, higher l-Met uptakes at 5 mM after pre-feeding with DL-Met and DL-HMTBA in JEJ, as well as a generally higher mRNA abundance of B^0^AT1. These physiological characteristics could be contributing factors to the more effective growth potential of male vs. female chickens.

## Declaration of competing interest

A. Lemme is an employee of Evonik Operations GmbH. This fact had no influence on the acquisition and interpretation of data. All other authors have no conflict of interest to declare.

## Appendix Supplementary materials

Image, application 1

## References

[bib0001] Amat C., Piqueras J.A., Planas J.M., Moretó M. (1999). Electrical properties of the intestinal mucosa of the chicken and the effects of luminal glucose. Poult. Sci..

[bib0002] Awad W.A., Aschenbach J.R., Setyabudi F.M., Razzazi-Fazeli E., Bohm J., Zentek J. (2007). In vitro effects of deoxynivalenol on small intestinal d-glucose uptake and absorption of deoxynivalenol across the isolated jejunal epithelium of laying hens. Poult. Sci..

[bib0003] Awad W.A., Böhm J., Razzazi-Fazeli E., Hulan H.W., Zentek J. (2004). Effects of deoxynivalenol on general performance and electrophysiological properties of intestinal mucosa of broiler chickens. Poult. Sci..

[bib0004] Awad W.A., Smorodchenko A., Hess C., Aschenbach J.R., Molnár A., Dublecz K., Khayal B., Pohl E.E., Hess M. (2015). Increased intracellular calcium level and impaired nutrient absorption are important pathogenicity traits in the chicken intestinal epithelium during colonization. Appl. Microbiol. Biotechnol..

[bib0005] Azahan E.A.E., Forbes J.M. (1989). Growth, food-intake and energy-balance of layer and broiler-chickens offered glucose in the drinking-water and the effect of dietary-protein content. Br. Poult. Sci..

[bib0006] Benyi K., Tshilate T.S., Netshipale A.J., Mahlako K.T. (2015). Effects of genotype and sex on the growth performance and carcass characteristics of broiler chickens. Trop. Anim. Health. Prod..

[bib0007] Broer A., Klingel K., Kowalczuk S., Rasko J.E., Cavanaugh J., Broer S. (2004). Molecular cloning of mouse amino acid transport system B0, a neutral amino acid transporter related to Hartnup disorder. J. Biol. Chem..

[bib0008] Cobb&Vantress (2022).

[bib0009] DLG Ausschuss Technik in der Tierhaltung R.A., Uhlenkamp A., Kämmerling D., Döhring S., Berk J., Grashorn M., Werner D., Mann K.H., Bös B., Mirbach D. (2018).

[bib0010] Drew M.D., Van Kessel A.G., Maenz D.D. (2003). Absorption of methionine and 2-hydroxy-4-methylthiobutoanic acid in conventional and germ-free chickens. Poult. Sci..

[bib0011] England A., Gharib-Naseri K., Kheravii S.K., Wu S.B. (2023). Influence of sex and rearing method on performance and flock uniformity in broilersdimplications for research settings. Anim. Nutrit..

[bib0012] Gesemann M., Lesslauer A., Maurer C.M., Schonthaler H.B., Neuhauss S.C. (2010). Phylogenetic analysis of the vertebrate excitatory/neutral amino acid transporter (SLC1/EAAT) family reveals lineage specific subfamilies. BMC Evol. Biol..

[bib0013] Gilbert E.R., Li H., Emmerson D.A., Webb K.E., Wong E.A. (2007). Developmental regulation of nutrient transporter and enzyme mRNA abundance in the small intestine of broilers. Poult. Sci..

[bib0014] Giurgea R., Coprean D., Domide A., Roman I. (1991). Thiourea and methionine effects upon glucose and leucine absorption in chicken jejunum. Rev. Roum. Physiol..

[bib0015] ISO 12099:2017 (2017).

[bib0016] Jando J., Camargo S.M.R., Herzog B., Verrey F. (2017). Expression and regulation of the neutral amino acid transporter B0AT1 in rat small intestine. PLoS. One..

[bib0017] Kaminski N.A., Wong E.A. (2018). Differential mRNA expression of nutrient transporters in male and female chickens. Poult. Sci..

[bib0018] Klünemann M., Romero L.F., Acman M., Milfort M.C., Fuller A.L., Rekaya R., Aggrey S.E., Payling L.M., Lemme A. (2024). Multitissue transcriptomics demonstrates the systemic physiology of methionine deficiency in broiler chickens. Animal.

[bib0019] Laurino P., Tawfik D.S. (2017). Spontaneous emergence of S-adenosylmethionine and the evolution of methylation. Angew. Chem. Int .Ed. Engl..

[bib0020] Lemme A., Hoehler D., Brennan J.J., Mannion P.F. (2002). Relative effectiveness of methionine hydroxy analog compared to DL-methionine in broiler chickens. Poult. Sci..

[bib0021] Lemme A., Li Z.Y., Dorigam J. (2024). Meta-analyses of methionine source concept validation trials in broilers. Animals.

[bib0022] Lemme A., Naranjo V., de Paula Dorigam J.C. (2020). Utilization of methionine sources for growth and met+cys deposition in broilers. Animals.

[bib0023] Lerner J., Karcher C.A. (1978). Kinetic-properties of an imino acid transport-system in chicken intestine. Comp. Biochem. Physiol..

[bib0024] Lerner J., Sattelme P., Rush R. (1975). Kinetics of methionine influx into various regions of chicken intestine. Comp. Biochem. Physiol..

[bib0025] Lindberg J.E. (2023). Review: nutrient and energy supply in monogastric food producing animals with reduced environmental and climatic footprint and improved gut health. Animal.

[bib0026] Llames C.R., Fontaine J. (1994). Determination of amino-acids in feeds - collaborative study. J. AOAC. Int..

[bib0027] Lopez K.P., Schilling M.W., Corzo A. (2011). Broiler genetic strain and sex effects on meat characteristics. Poult. Sci..

[bib0028] Maenz D.D., EngeleSchaan C.M. (1996). Methionine and 2-hydroxy-4-methylthiobutanoic acid are partially converted to nonabsorbed compounds during passage through the small intestine and heat exposure does not affect small intestinal absorption of methionine sources in broiler chicks. J. Nutrit..

[bib0029] Mastrototaro L., Sponder G., Saremi B., Aschenbach J.R. (2016). Gastrointestinal methionine shuttle: priority handling of precious goods. IUBMB. Life..

[bib0030] McWhorter T.J., Green A.K., Karasov W.H. (2010). Assessment of radiolabeled d-glucose and the nonmetabolizable analog 3-O-methyl-d-glucose as tools for In vivo absorption studies. Physiol. Biochem. Zool..

[bib0031] Miles R.D., Butcher G.D., Henry P.R., Littell R.C. (2006). Effect of antibiotic growth promoters on broiler performance, intestinal growth parameters, and quantitative morphology. Poult. Sci..

[bib0032] Miska K.B., Fetterer R.H. (2019). Expression of amino acid and sugar transporters, aminopeptidase, and the di- and tri-peptide transporter PepT1; differences between modern fast growing broilers and broilers not selected for rapid growth. Poult. Sci..

[bib0033] Mitchell M.A., Lemme A. (2008). Examination of the composition of the luminal fluid in the small intestine of broilers and absorption of amino acids under various ambient temperatures measured In vivo. Int. J. Poult. Sci..

[bib0034] Mitchell M.A., Levin R.J. (1981). Amino acid absorption in jejunum and ileum in vivo – a kinetic comparison of function on surface area and regional bases. Experentia.

[bib0035] Moreto M., Planas J.M. (1989). Sugar and amino-acid-transport properties of the chicken Ceca. J. Exp. Zool..

[bib0036] Nickel A., Kottra G., Schmidt G., Danier J., Hofmann T., Daniel H. (2009). Characteristics of transport of selenoamino acids by epithelial amino acid transporters. Chem. Biol. Interact..

[bib0037] Palacin M. (1994). A new family of proteins (rBAT and 4F2hc) involved in cationic and zwitterionic amino acid transport: a tale of two proteins in search of a transport function. J. Exp. Biol..

[bib0038] Riesenfeld G., Sklan D., Bar A., Eisner U., Hurwitz S. (1980). Glucose-absorption and starch digestion in the intestine of the chicken. J. Nutrit..

[bib0039] Romanet S., Aschenbach J.R., Pieper R., Zentek J., Htoo J.K., Whelan R.A., Mastrototaro L. (2020). Dietary supplementation of dl-methionine potently induces sodium-dependent l-methionine absorption in Porcine Jejunum ex vivo. J. Nutrit..

[bib0040] Romanet S., Aschenbach J.R., Pieper R., Zentek J., Htoo J.K., Whelan R.A., Mastrototaro L. (2021). Expression of proposed methionine transporters along the gastrointestinal tract of pigs and their regulation by dietary methionine sources. Genes Nutr..

[bib0041] Romanet S., Schermuly I.I., Lemme A., Zentek J., Aschenbach J.R. (2025). Optimizing the incubation solution for ex vivo investigations on intestinal tissues of chickens. Proc. Soc. Nutr. Physiol..

[bib0042] Ruan T., Li L., Lyu Y., Luo Q., Wu B. (2018). Effect of methionine deficiency on oxidative stress and apoptosis in the small intestine of broilers. Acta. Vet. Hung..

[bib0043] Sklan D., Geyra A., Tako E., Gal-Gerber O., Uni Z. (2003). Ontogeny of brush border carbohydrate digestion and uptake in the chick. Brit. J. Nutr..

[bib0044] Stein E.D., Chang S.D., Diamond J.M. (1987). Comparison of different dietary amino-acids as inducers of intestinal amino-acid-transport. Am. J. Physiol..

[bib0045] To V., Masagounder K., Loewen M.E. (2021). Critical transporters of methionine and methionine hydroxyl analogue supplements across the intestine: what we know so far and what can be learned to advance animal nutrition. Comparat. Biochem. Physiol. a-Molecul. Integrat. Physiol..

[bib0046] Torras-Llort M., Torrents D., Soriano-García J.P., Gelpí J.L., Estévez R., Ferrer R., Palacín M., Moretó M. (2001). Sequential amino acid exchange across b-like system in chicken brush border jejunum. J. Membr. Biol..

[bib0047] Vandesompele J., De Preter K., Pattyn F., Poppe B., Van Roy N., De Paepe A., Speleman F. (2002). Accurate normalization of real-time quantitative RT-PCR data by geometric averaging of multiple internal control genes. Genom. Biol..

[bib0048] Vazquez-Anon M., Gonzalez-Esquerra R., Saleh E., Hampton T., Ritcher S., Firman J., Knight C.D. (2006). Evidence for 2-hydroxy-4(methylthio) butanoic acid and DL-methionine having different dose responses in growing broilers. Poult. Sci..

[bib0049] Vieira S.L., Lemme A., Goldenberg D.B., Brugalli I. (2004). Responses of growing broilers to diets with increased sulfur amino acids to lysine ratios at two dietary protein levels. Poult. Sci..

[bib0050] Vinardell M.P., Lopera M.T., Moreto M. (1986). Absorption of 3-oxy-methyl-d-glucose by chicken cecum and jejunum in vivo. Comp. Biochem. Physiol. A. Comp. Physiol..

[bib0051] Wang J.L., Dong Y., Grewer C. (2022). Functional and kinetic comparison of Alanine Cysteine serine transporters ASCT1 and ASCT2. Biomolecules.

[bib0052] Wang X., Peebles E.D., Morgan T.W., Harkess R.L., Zhai W. (2015). Protein source and nutrient density in the diets of male broilers from 8 to 21 d of age: effects on small intestine morphology. Poult. Sci..

[bib0053] Wang Y.L., Yin X.N., Yin D.F., Lei Z., Mahmood T., Yuan J.M. (2019). Antioxidant response and bioavailability of methionine hydroxy analog relative to DL-methionine in broiler chickens. Anim Nutrit..

[bib0054] Weintraut M.L., Kim S., Dalloul R.A., Wong E.A. (2016). Expression of small intestinal nutrient transporters in embryonic and posthatch turkeys. Poult. Sci..

[bib0055] Wu G., Fang Y.Z., Yang S., Lupton J.R., Turner N.D. (2004). Glutathione metabolism and its implications for health. J. Nutr..

[bib0056] Yodseranee R., Bunchasak C. (2012). Effects of dietary methionine source on productive performance, blood chemical, and hematological profiles in broiler chickens under tropical conditions. Trop. Anim. Health. Prod..

[bib0057] Zhang S., Saremi B., Gilbert E.R., Wong E.A. (2017). Physiological and biochemical aspects of methionine isomers and a methionine analogue in broilers. Poult. Sci..

[bib0058] Zhang Y., Zhuang Z., Mahmood T., Mercier Y., Jin Y., Huang X., Li K., Wang S., Xia W., Wang S., Yu M., Chen W., Zheng C. (2023). Dietary supplementation with 2-hydroxy-4-methyl(thio) butanoic acid and DL-methionine improves productive performance, egg quality and redox status of commercial laying ducks. Anim. Nutrit..

