# Supplementary material for: Transport and expression of transporters for 3-O-methyl-D-glucose and L-methionine along the intestine of broiler chickens receiving different methionine supplements
Source: Poult Sci. 2025 Apr 7;104(6):105142. doi: 10.1016/j.psj.2025.105142 (PMC12018188; doi:10.1016/j.psj.2025.105142)
Supplement: Supplementary file 1 [file mmc1.docx]

**Supplementary Material**

**Supplementary table 1: *P*-values of all performed three-Way ANOVA for 3-OMG and L-Met**

|  | 3-OMG | | L-Met | |
| --- | --- | --- | --- | --- |
| Factors | **50 µM** | **5 mM** | **50 µM** | **5 mM** |
| Tissue | < 0.001 | < 0.001 | < 0.001 | < 0.001 |
| Sodium | <0.001 | 0.003 | 0.055 | 0.62 |
| Diet | 0.87 | 0.15 | 0.74 | 0.12 |
| Tissue × sodium | < 0.001 | < 0.001 | 0.021 | 0.47 |
| Tissue × diet | 0.54 | 0.65 | 0.74 | 0.58 |
| Sodium × diet | 0.92 | 0.27 | 0.96 | 0.23 |
| Tissue × sodium × diet | 0.89 | 0.69 | 0.90 | 0.45 |

|  | 3-OMG | | L-Met | |
| --- | --- | --- | --- | --- |
| Factors | **50 µM** | **5 mM** | **50 µM** | **5 mM** |
| Sex | 0.31 | 0.36 | 0.096 | 0.67 |
| Diet | 0.92 | 0.25 | 0.67 | 0.14 |
| Tissue | < 0.001 | < 0.001 | < 0.001 | < 0.001 |
| Sex × Diet | 0.18 | 0.52 | 0.19 | 0.50 |
| Sex × tissue | 0.021 | 0.64 | 0.011 | 0.75 |
| Diet × tissue | 0.59 | 0.73 | 0.77 | 0.64 |
| Sex × diet × tissue | 0.62 | 0.27 | 0.49 | 0.024 |

|  | 3-OMG | | L-Met | |
| --- | --- | --- | --- | --- |
| Factors | **50 µM** | **5 mM** | **50 µM** | **5 mM** |
| Diet | 0.97 | 0.68 | 0.92 | 0.67 |
| Sodium | 0.044 | 0.069 | 0.39 | 0.78 |
| Sex | 0.40 | 0.36 | 0.28 | 0.52 |
| Diet × sodium | 0.93 | 0.56 | 1.00 | 0.51 |
| Diet × sex | 0.75 | 0.77 | 0.83 | 0.89 |
| Sodium × sex | 0.42 | 0.80 | 0.62 | 0.85 |
| Diet × sodium × sex | 0.92 | 0.77 | 0.94 | 0.79 |

|  | 3-OMG | | L-Met | |
| --- | --- | --- | --- | --- |
| Factors | **50 µM** | **5 mM** | **50 µM** | **5 mM** |
| Tissue | < 0.001 | < 0.001 | < 0.001 | < 0.001 |
| Sodium | < 0.001 | 0.005 | 0.057 | 0.58 |
| Sex | 0.29 | 0.29 | 0.10 | 0.63 |
| Tissue × sodium | < 0.001 | < 0.001 | 0.016 | 0.38 |
| Tissue × sex | 0.011 | 0.66 | 0.008 | 0.81 |
| Sodium × sex | 0.19 | 0.46 | 0.34 | 0.61 |
| Tissue × sodium × sex | 0.95 | 0.62 | 0.93 | 0.61 |
